# Experimental data from strengthening bamboo reinforcement using adhesives and hose-clamps

**DOI:** 10.1016/j.dib.2019.104827

**Published:** 2019-11-16

**Authors:** Muhtar

**Affiliations:** Department of Civil Engineering, Faculty of Engineering, University of Muhammadiyah Jember, Jember, 68121, Indonesia

**Keywords:** Bond strength, Bamboo reinforcement, Strengthening, Bamboo

## Abstract

The bamboo treatment process starts with cutting, soaking in water, draining in free air, reinforcing in the fireplace, first-stage adhesive coating, hose-clamp installation, second-stage adhesive coating, and sand resurfacing. Data was taken from experimental testing of bamboo materials and bond strength tests of bamboo reinforcement in the laboratory of the Faculty of Engineering, University of Brawijaya Malang. The aim of treating and strengthening bamboo reinforcement is to overcome low-load capacity and prevent collapse due to slippage in bamboo reinforced concrete elements. Adhesive coating is employed to increase durability and prevent water absorption, while installing hose-clamps increases bamboo reinforcement slip resistance. The process outlined here represents the way to approach bamboo reinforcement, and laboratory data is processed into graphic images and tables of bond strength of bamboo reinforcement providing the basis for further research. This article comprises a standard operating procedure for treatment of bamboo reinforcement, graphic images, documentation photos, and data tables. The data is related to “Enhancing bamboo reinforcement using a hose-clamp to increase bond-stress and slip resistance” [1].

**Table:** Specifications Table

Subject	Engineering
Specific subject area	Civil and Structural Engineering
Type of data	Table, image
How data were acquired	Data was obtained from two experimental tests, namely the pull-out test (Fig. 14) and the beam flexural test (Fig. 16). Then, the test data is processed and analyzed into table data, image data, and documentation data
Data format	Raw and analyzed
Parameters for data collection	The main requirement for using bamboo as a concrete reinforcement is initial treatment. This involves the use of adhesives and hose-clamps to effectively increase the bond strength of bamboo reinforcement. Therefore a standard operating procedure and test data need to be established for the development of further research
Description of data collection	Bond strength data was obtained from testing bamboo reinforcement specimens after different treatments. Data from each specimen is processed and analyzed into table data, image data, and photo data which are described as single data. This single data is collected together, processed, compared, re-analyzed into table data, image data, and photo data, which is then called intact data
Data source location	University of Muhammadiyah Jember, Jember, 68121, Indonesia, and University of Brawijaya, Malang 65145, Indonesia
Data accessibility	Data with the article, raw data can be found in Table 1, http://bit.ly/2PvYuXS, and http://bit.ly/2NxiYgq
Related research article	Enhancing bamboo reinforcement using a hose-clamp to increase bond-stress and slip resistance. https://doi.org/10.1016/j.jobe.2019.100896 [1],

**Table:** 

**Value of the Data** • This data is useful as scientific evidence about treatment methods for bamboo used as concrete reinforcement. • This data benefits researchers and rural communities with an abundance of bamboo. • This data includes several bamboo treatment methods that can be used as a basis for further research development. • The data on the effects of adhesives and hose-clamps on bond strength can be used as a basis for further research, especially on tensile elements such as truss, length of distribution, etc. • The added value of this data is that it encourages new efforts in the strengthening of bamboo reinforcement using adhesives and hose clamps for concrete reinforcement, using renewable and low-cost materials to empower poor communities in disadvantaged village areas – especially bamboo farming communities.

## 1 Data

In this article, data is presented in the form of the standard operating procedure (SOP) for bamboo reinforcement treatment, graphic images, documentation photos, and data tables. Standard operating procedure (SOP) of bamboo reinforcement treatment starts with cutting, soaking in water, draining in free air, reinforcing in the fireplace, first-stage adhesive coating, hose-clamp installation, second-stage adhesive coating, and sand resurfacing as shown in Fig. 4, Fig. 5, Fig. 6, Fig. 7, Fig. 8, Fig. 9, Fig. 10, Fig. 11.

**Fig. 1:**
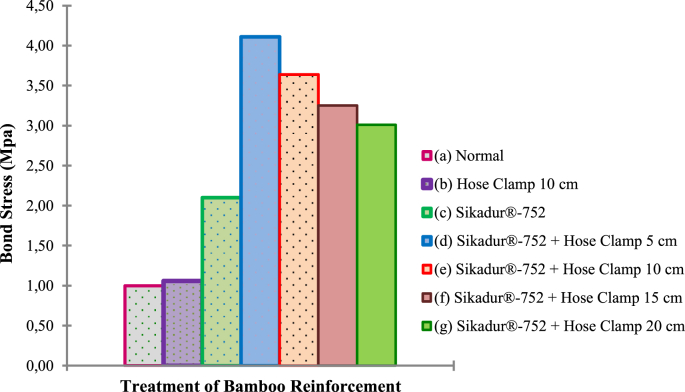
The data graph of bond stress of bamboo reinforcement.

**Fig. 2:**
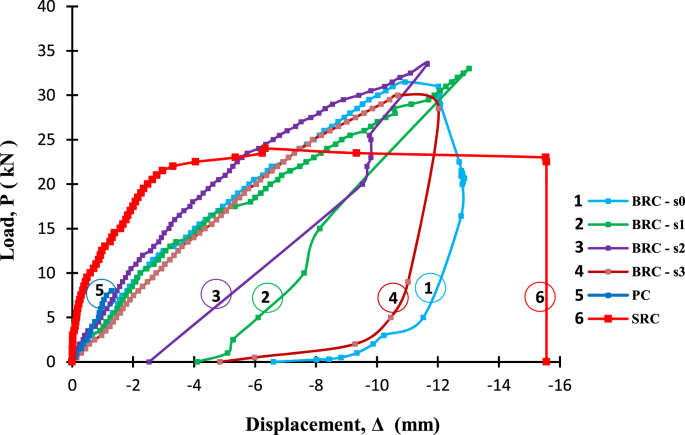
The load-deflection relationship of BRC beams and the readings of LVDT after the beam collapse.

**Fig. 3:**
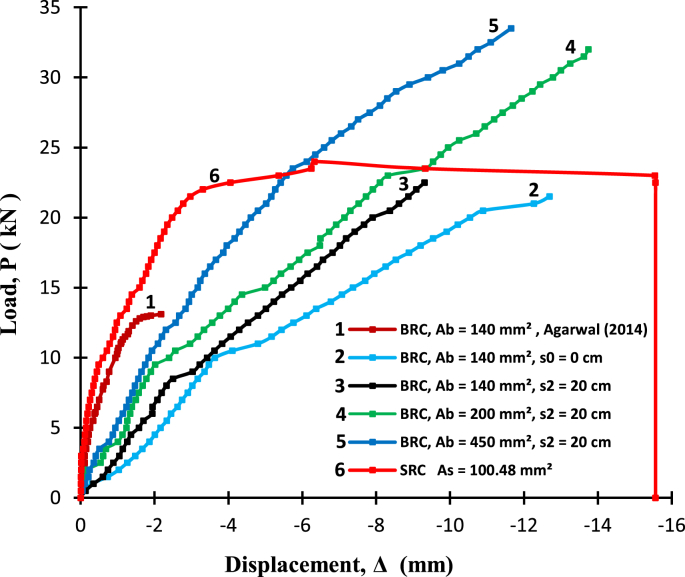
The load-deflection relationship of BRC beams compared to previous research results [2].

**Fig. 4:**
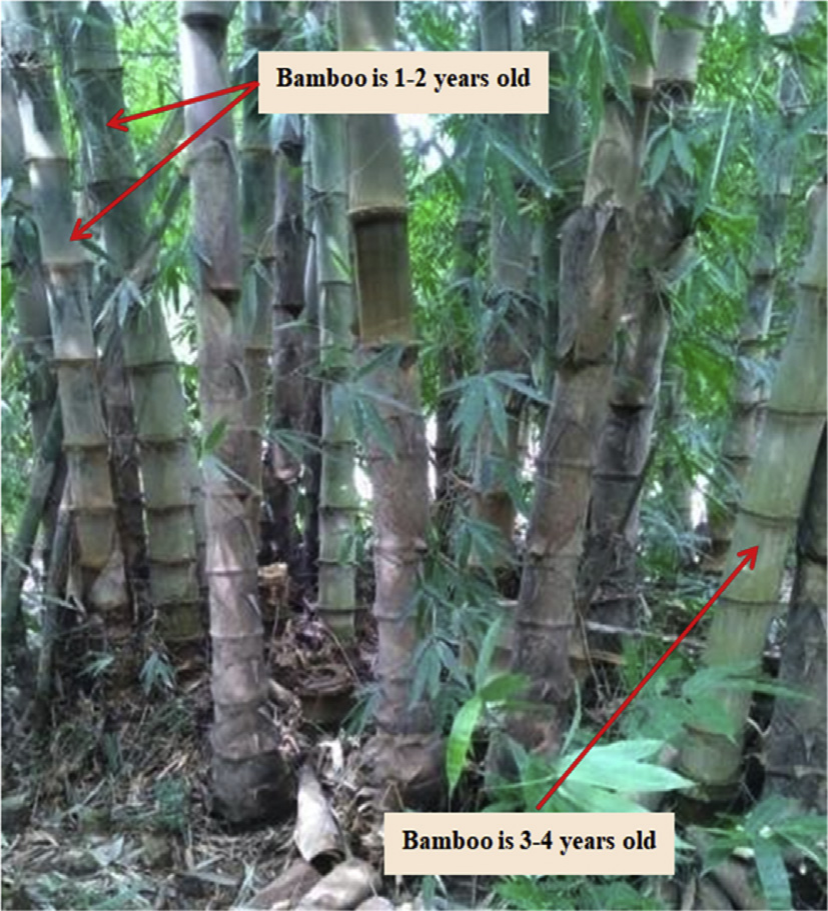
Bamboo petung (Dendrocalamus asper) at the felling site.

**Fig. 5:**
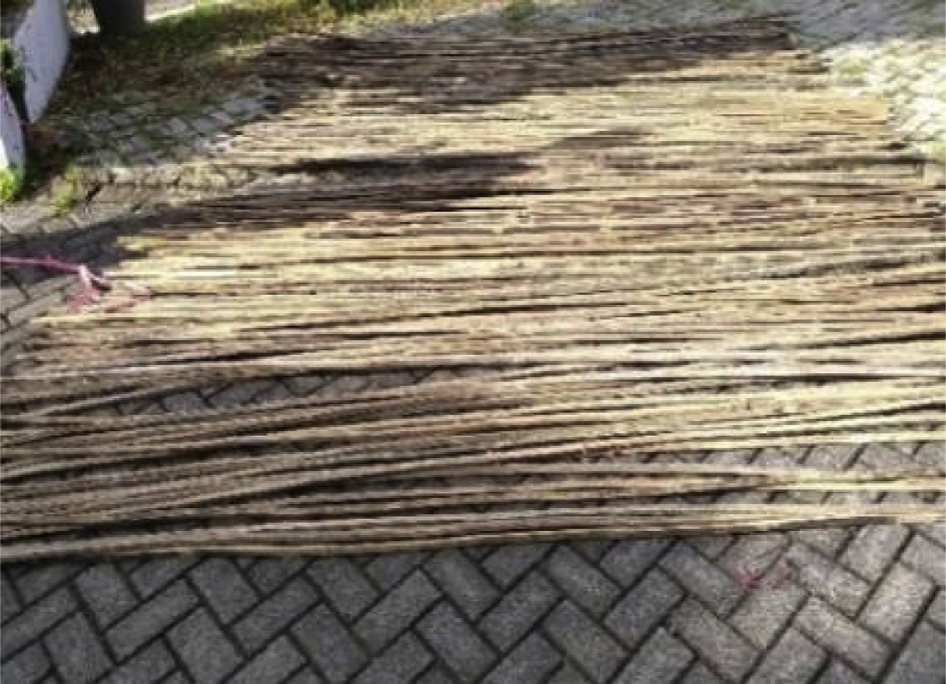
Drying of bamboo in free air for ±1 month after soaking.

**Fig. 6:**
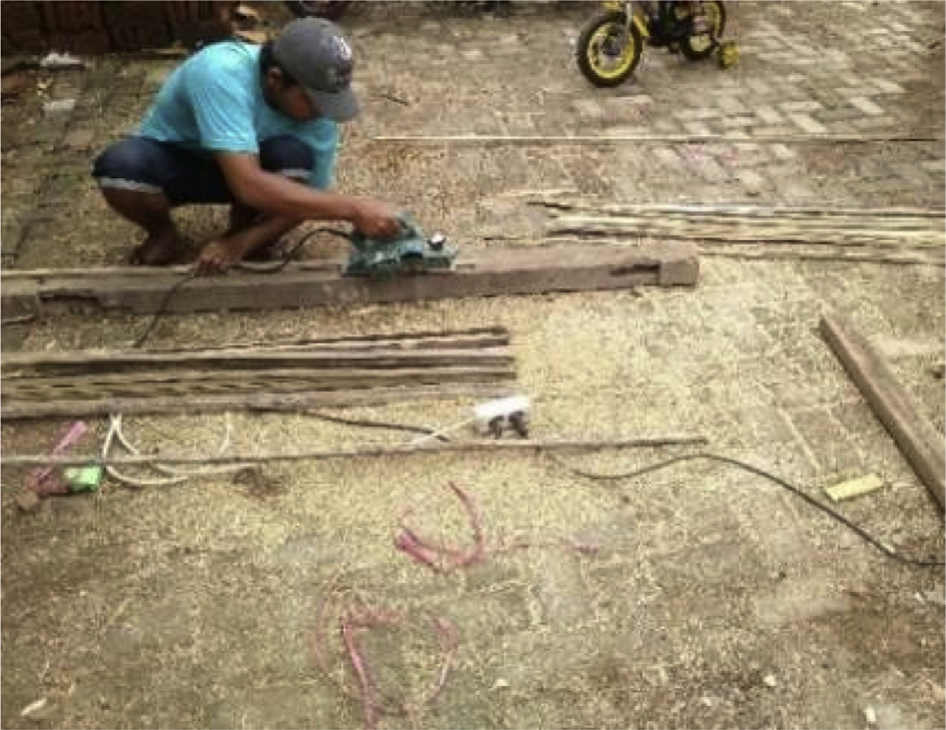
Exfoliating the inside of the bamboo and adjusting the dimensions of the bamboo to the reinforcement plan.

**Fig. 7:**
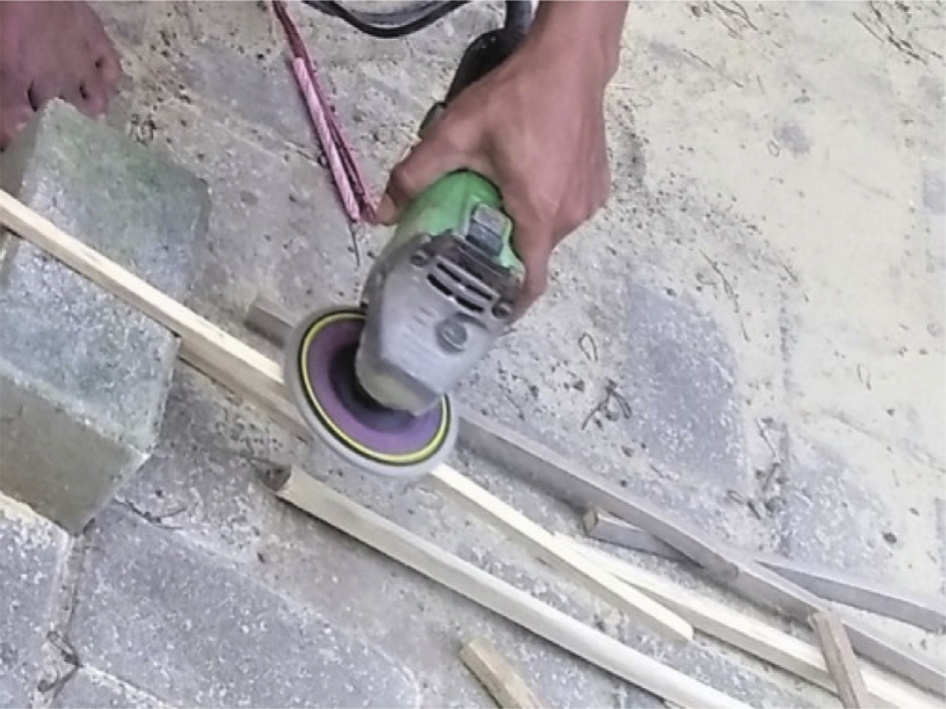
Fireplace; treating the surface of bamboo reinforcement with a grinding machine.

**Fig. 8:**
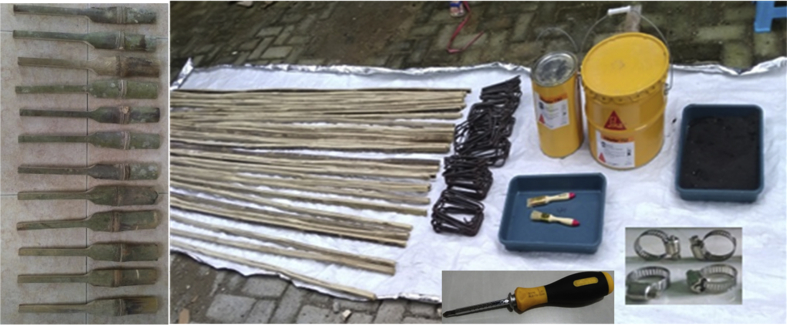
Bamboo reinforcement of bamboo, Sikadur®-752, fine sand, hose-clamps, brushes, and steel stirrups.

**Fig. 9:**
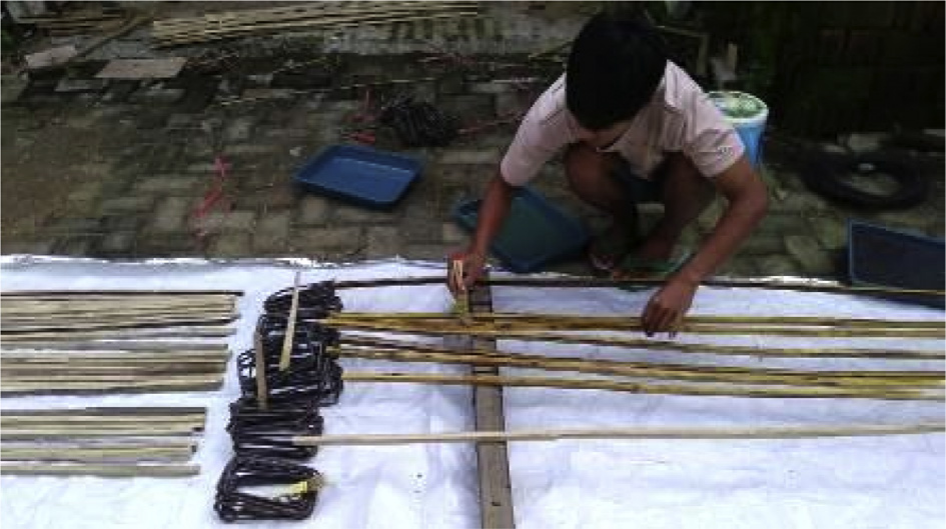
The initial surfacing of Sikadur®-752 adhesive.

**Fig. 10:**
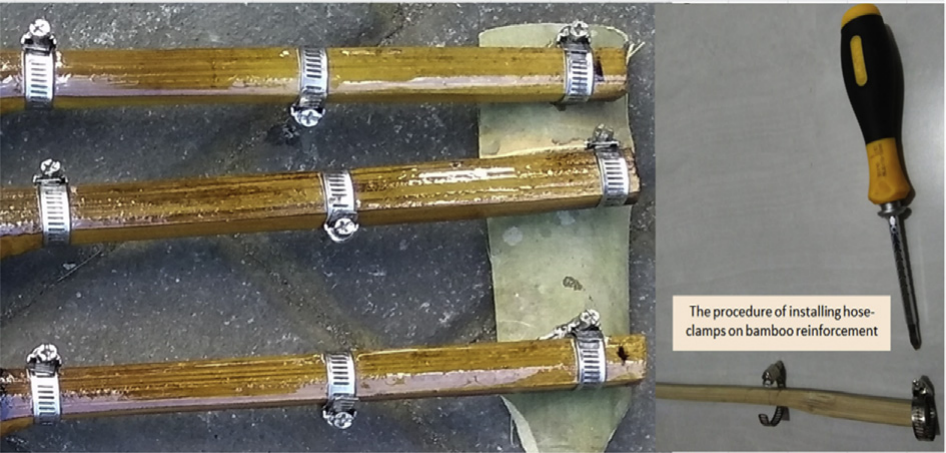
The installation of hose-clamps and the second layer of Sikadur®-752 adhesive.

**Fig. 11:**
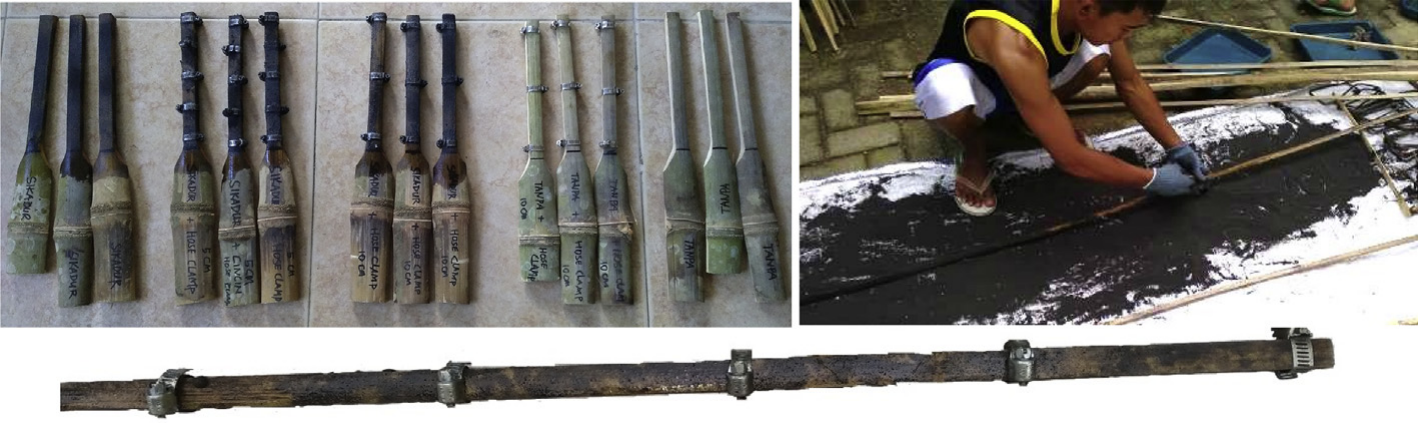
Fine sand surfacing (volcanic dust from Raung Mountain, Jember, Indonesia).

Raw data of bond stresses and failure patterns in bamboo reinforcement pull-out tests of seven treatments are presented in Table 1, while analysis data in the form of a graph of the relationship between bond stress and variations in bamboo reinforcement treatment is shown in Fig. 1. A data graph of the load-deflection relationship of a bamboo reinforced concrete beam, with LVDT readings from after the collapse of the beam, is shown in Fig. 2. The raw data from Fig. 2 is provided in the following link: http://bit.ly/2PvYuXS. Fig. 3 shows the load-deflection relationship of bamboo reinforced concrete beams compared to results from previous researchers. The raw data from Fig. 3 is shown in the following link: http://bit.ly/2NxiYgq.

**Table 1:** The data of bond strength and the failure pattern.

Sample no	Specimens of pull-out test	Wide b (mm)	Thick t (mm)	Depth embedded in concrete cylinders (mm)	The length of the circumference of the reinforcement (mm)	Tensile load (kN)	Bond strength (MPa)	Average bond strength (MPa)	Failure pattern
1	(a) Normal	15	15	200	60	12	1,00	1	bond-slip failure
2			15		60	12	1,00		
3			10		50	10	1,00		
4	(b) Hose Clamp 10 cm	15	15	200	60	13	1,08	1,09	bond-slip failure
5			15		60	13	1,08		
6			10		50	11	1,10		
7	(c) Sikadur®-752	15	15	200	60	31	2,58	2,5	bond-slip failure
8			15		60	30	2,50		
9			15		60	29	2,42		
10	(d) Sikadur®-752 + Hose Clamp 5 cm	15	15	200	60	49	4,08	4,11	bond and concrete cone failure
11			15		60	49	4,08		
12			15		60	50	4,17		
13	(d) Sikadur®-752 + Hose Clamp 10 cm	15	15	200	60	42	3,50	3,64	bond and concrete cone failure
14			15		60	44	3,67		
15			15		60	45	3,75		
16	(d) Sikadur®-752 + Hose Clamp 15 cm	15	15	200	60	39,5	3,29	3,14	bond and concrete cone failure
17			15		60	36	3,00		
18			15		60	37,5	3,13		
19	(d) Sikadur®-752 + Hose Clamp 20 cm	15	15	200	60	35	2,92	3,01	bond and concrete cone failure
20			15		60	36	3,00		
21			15		60	37,5	3,13		

## 2 Experimental design, materials, and methods

Bamboo petung (Dendrocalamus asper), features purplish black bamboo shoots, covered with feathers (miang) which resemble brown to black velvet. The large vertebra are 40–50cm long, and 12–18cm in diameter. Overall, petung bamboo reaches 20 m in height, with a curved tip, with color varying from green, dark green, purplish green, whitish green, or with white spots because of lichen. The nodes are surrounded by aerial roots. The thickness of the bamboo wall is between 11 and 36mm (Brink, M, 2008) in Wikipedia Indonesia [3].

Standard operating procedure (SOP) for preparing bamboo reinforcement includes installing hose-clamps, waterproof coating, sand coating, pull-out test setting, and flexural test setting.

Step 1: Bamboo cutting.

The bamboo used was at least three years old, with white spots, as shown in Fig. 4. Bamboo stems used are 6 m long from their base. This is because stems exhibit stronger mechanical properties and thickness up to 6 m. After logging, bamboo is cut to the size planned, and soaked in water for more than a month. Bamboo reinforcement pieces are cut to a length of approximately 1100 × 15mm. The number of nodes varies between two and three pieces.

Step 2: Drying of bamboo in free air for ±1 month [1,2,4] after soaking in water, as shown in Fig. 5.

Step 3: Fireplace treatment and adjustment of dimensions, as shown in Fig. 6.

Step 4: Fireplace and treating the surface of the bamboo reinforcement with a grinding machine, as shown in Fig. 7.

Step 5: Preparation of materials and tools, including, Sikadur®-752, fine sand, hose-clamps, and brushing, as shown in Fig. 8.

Step 6: The initial coating of adhesive Sikadur®-752, and installation of hose-clamps on the bamboo reinforcement, as shown in Figs. 9 and 10.

Instructions for installing hose-clamps:

• The initial stage of Sikadur®-752 adhesive surfacing is carried out after the fireplace treatment, based on the planned dimensions.
• Sikadur®-752 surfacing is carried out until it is even.
• Installation of hose-clamps is carried out after the first Sikadur®-752 layer is dry.
• The hose-clamps are installed in two ways, namely (1) by loosening the nut-bolts of the hose-clamps and directly inserting from the end of the bamboo reinforcement, or (2) opening the hose-clamps and installing them at the point of the installation plan.
• Hose-clamps are tightened with a screwdriver, turned until it stops. There should be no additional tightening, so as to avoid defects and waterproof coating leaks on the bamboo reinforcement.
• After the clamps are installed, a second Sikadur®-752 surfacing is carried out.
• The coating of sand on bamboo reinforcement is applied after the second layer of Sikadur®-752 adhesive is half dry, as shown in Fig. 11.

**Fig. 12:**
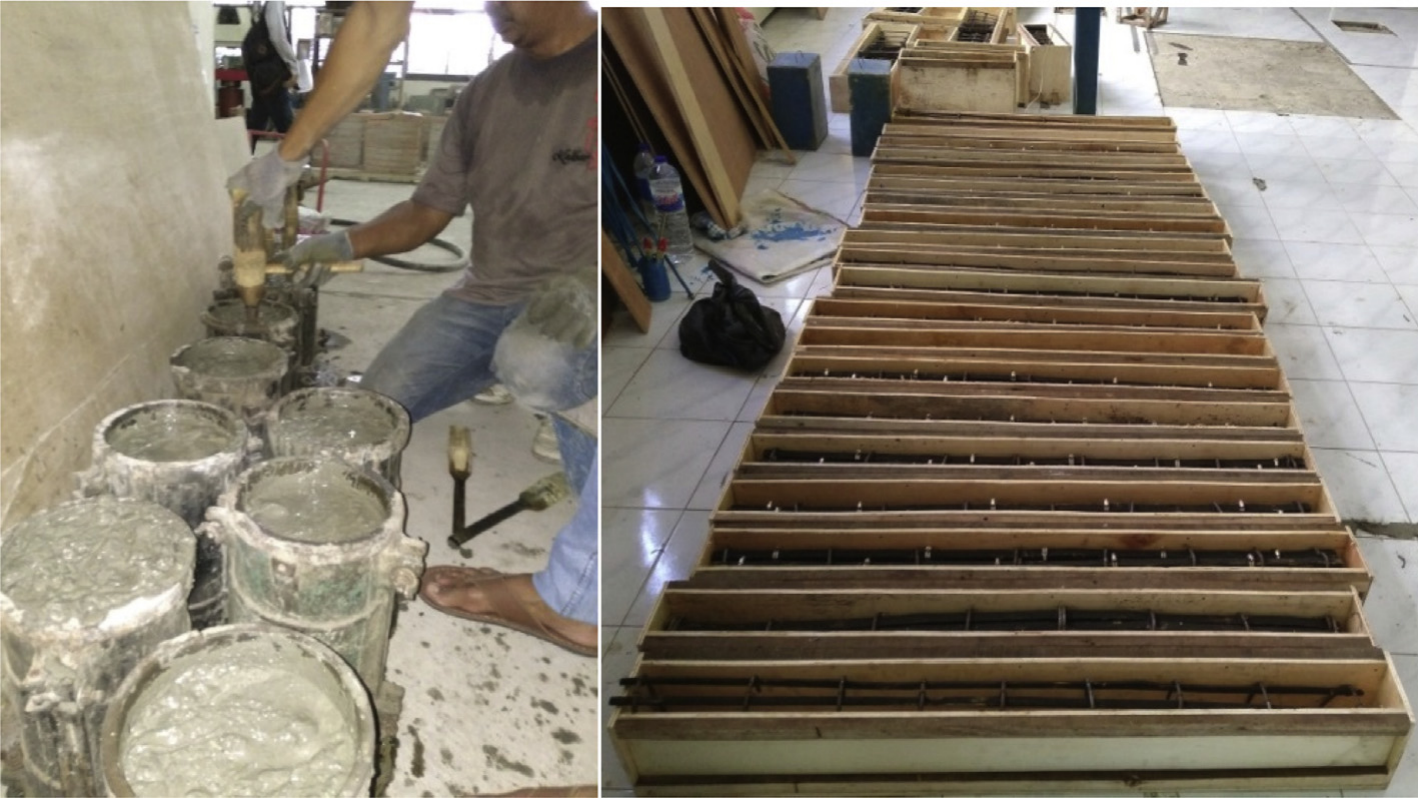
Preparing pull-out test and flexural test specimens.

Step 7: The construction of pull-out test specimens and flexural test specimens of bamboo reinforcement, as shown in Fig. 12.

**Fig. 13:**
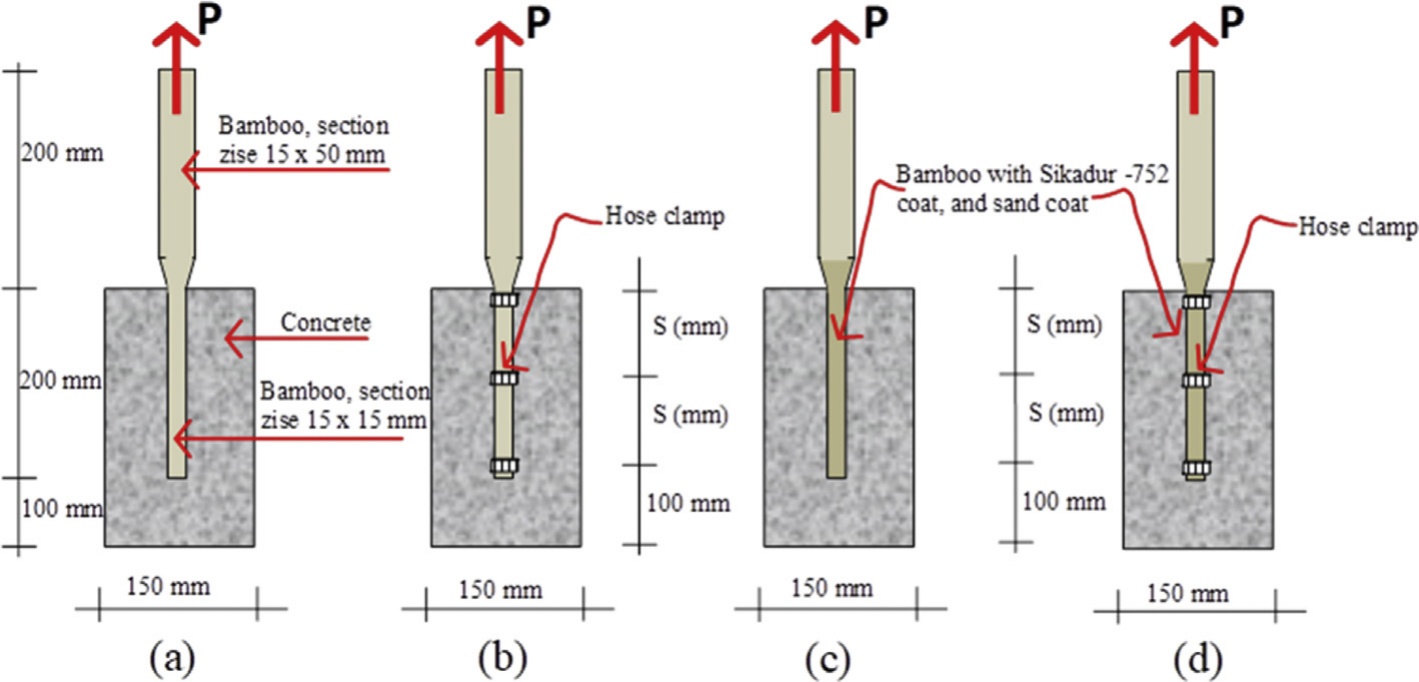
Specimen details for the pull-out test.

**Fig. 14:**
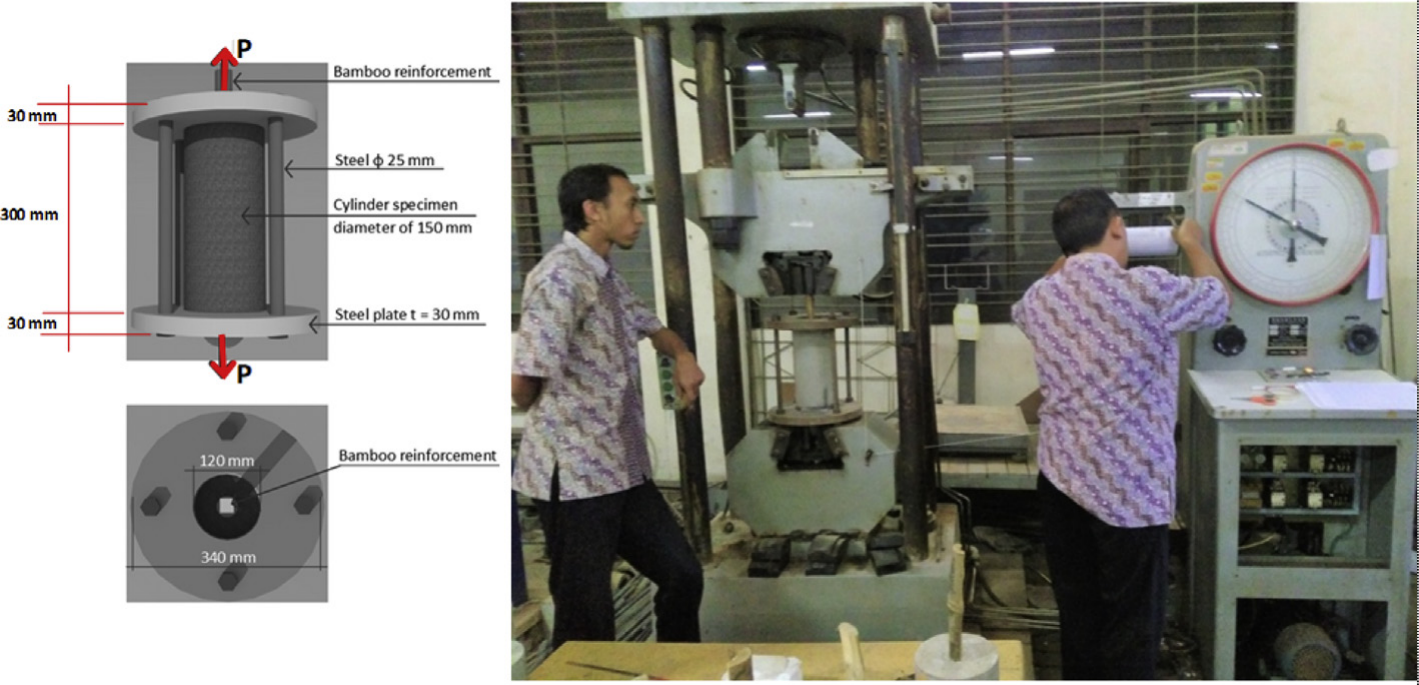
Pull-out test settings.

Step 8: Pull-out test setting. The bamboo reinforcement bond strength test uses a conventional pull-out test method [5]. Specimen details and pull-out test settings are shown in Fig. 13 and Fig. 14.

**Fig. 15:**
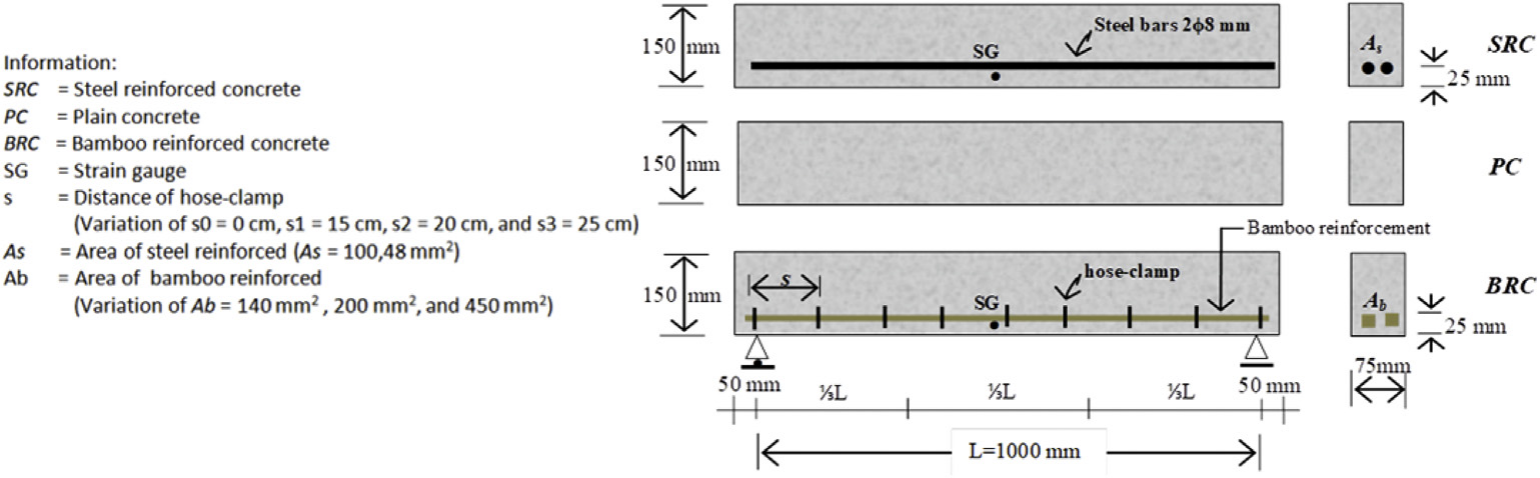
Detail and geometry of the bamboo reinforced concrete beam.

**Fig. 16:**
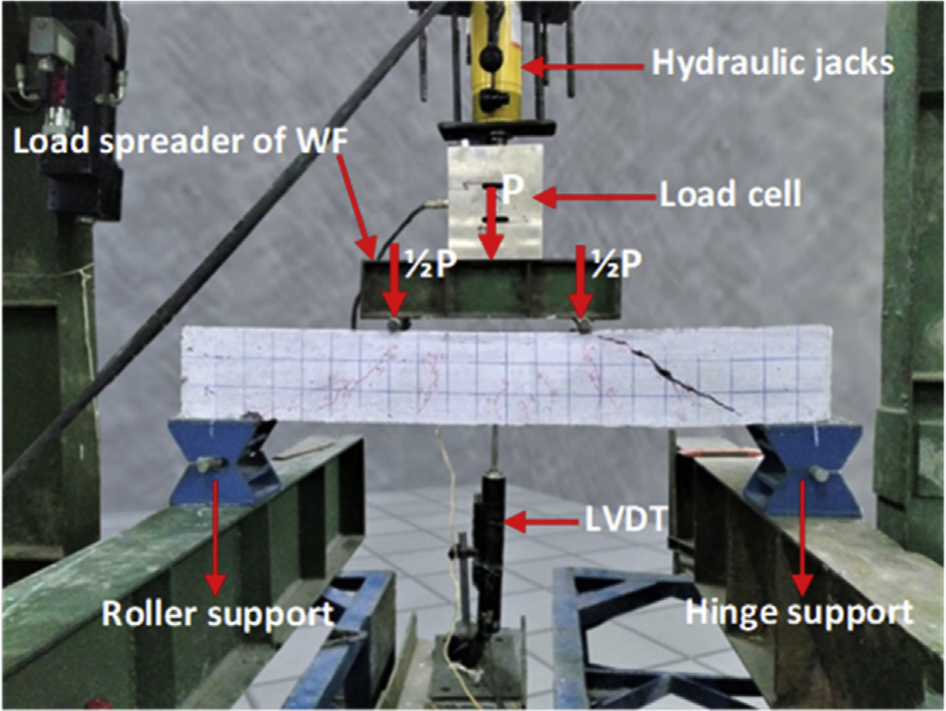
Flexural test settings for the four-point flexural test method.

Step 9: The flexural test setting. This employs the four-point flexural test method. Details, geometry, and flexural test settings are shown in Fig. 15 and Fig. 16.
